# Suppressing PEDOT:PSS Doping-Induced Interfacial Recombination Loss in Perovskite Solar Cells

**DOI:** 10.1021/acsenergylett.1c02577

**Published:** 2022-01-06

**Authors:** Yi-Chun Chin, Matyas Daboczi, Charlie Henderson, Joel Luke, Ji-Seon Kim

**Affiliations:** Department of Physics and Center for Processable Electronics, Imperial College London, London SW7 2AZ, U.K.

## Abstract

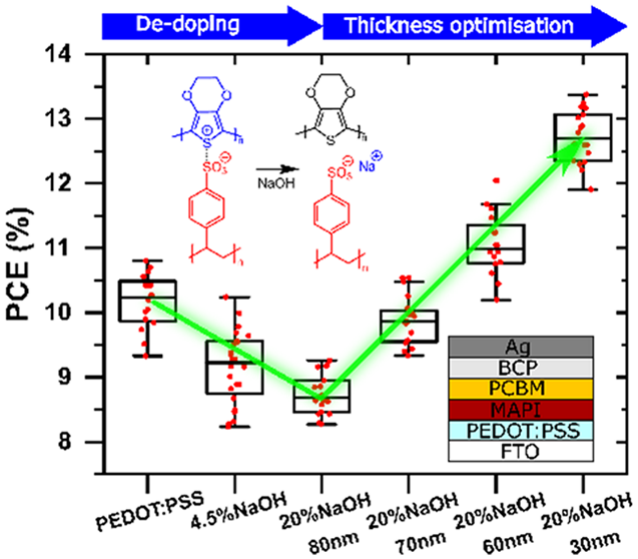

PEDOT:PSS is widely used as a hole transport layer (HTL) in perovskite solar cells (PSCs) due to its facile processability, industrial scalability, and commercialization potential. However, PSCs utilizing PEDOT:PSS suffer from strong recombination losses compared to other organic HTLs. This results in lower open-circuit voltage (*V*_OC_) and power conversion efficiency (PCE). Most studies focus on doping PEDOT:PSS to improve charge extraction, but it has been suggested that a high doping level can cause strong recombination losses. Herein, we systematically dedope PEDOT:PSS with aqueous NaOH, raising its Fermi level by up to 500 meV, and optimize its layer thickness in p-i-n devices. A significant reduction of recombination losses at the dedoped PEDOT:PSS/perovskite interface is evidenced by a longer photoluminescence lifetime and higher magnitude of surface photovoltage, leading to an increased device *V*_OC_, fill factor, and PCE. These results provide insights into the relationship between doping level of HTLs and interfacial charge carrier recombination losses.

In the past decade, perovskite solar cells (PSCs) have become one of the most-promising next-generation solar cell technologies with sharply increasing power conversion efficiencies (PCEs) of up to 25.5% together with improved operational lifetimes (∼1000 h).^1,2^ Charge transport layers and their development play an important role in PSCs, as they govern charge extraction and can contribute toward device degradation.^3−6^ Particularly, hole transport layers (HTLs) have been shown to be critical in determining both the PCE and the lifetime of PSCs.^7,8^ The highest-performing HTLs are organic materials that have lower hole mobilities compared to inorganic perovskites. This mobility mismatch in most cases results in charge accumulation at the interface between perovskite and HTL, eventually leading to undesired recombination losses.^9−11^ In addition to low mobility, trap states at the perovskite/HTL interface can significantly reduce device performance by acting as recombination sites, reducing open-circuit voltage (*V*_OC_).^12−14^ In order to achieve higher PCE, strategies for alleviating these issues and fundamental understanding of the mechanisms behind them are important and still need full attention.

One common strategy used to minimize the critical issues mentioned above is to add p-type dopants to the HTL.^15,16^ The p-type dopants deepen the Fermi level (*E*_F_) of the HTL and, therefore, can create favorable interfacial energetic alignment, which reduces the energy loss at the interface between the perovskite and HTL.^17−19^ Importantly, p-type dopants increase the conductivity of the HTL, which can improve the fill factor (FF) of the devices *via* more-efficient charge extraction.^20−23^ Despite the success of utilizing dopants in the HTL, some undesirable effects are also introduced with doping. One issue is the device instability caused by dopant dissociation and the hygroscopic nature of some dopants such as lithium(bis(trifluoromethanesulfonyl)imide (LiTFSI).^24,25^ Another significant effect of doping the HTL is strong recombination loss at the interface. One previous study has shown that under highly doped conditions, the pre-existing high density of holes in the HTL can nonradiatively recombine with the photoexcited electrons in the perovskite layer.^9^ Such nonradiative losses lead to reduced *V*_OC_ and PCE in solar cells. Although strong recombination loss is clearly related to the high doping level in HTL, there is still a lack of evidence as to whether dedoping the HTL can reduce recombination losses at the interface and, furthermore, improve device performance. Therefore, a systematic study on dedoping of a highly doped HTL such as PEDOT:PSS can lead to a better understanding of the loss mechanisms and provide a wider strategy to HTL modification.

PEDOT:PSS is one of the most widely studied HTLs in perovskite devices because of its facile fabrication and suitable wettability for perovskite formation on top.^26^ Also, PEDOT:PSS is well-adopted in industry with a very low price and high quality, which makes it a strong HTL candidate for PSC commercialization. However, its highly doped nature leads to the issues mentioned above (such as strong interfacial recombination) rendering PEDOT:PSS as an HTL with relatively low stability and low performance compared to other HTLs for PSCs.^9,27,28^ Many studies have followed the common approach of further p-doping of PEDOT:PSS in order to match the energetics and to increase conductivity for performance improvements.^17−19,29^ In this work, instead of doping PEDOT:PSS, we provide a strategy to investigate and reduce interfacial recombination by dedoping PEDOT:PSS. Since dedoping decreases the conductivity, an efficient charge extraction is maintained by reducing the thickness of dedoped PEDOT:PSS layer. This study provides the clear linkage between the HTL doping level and interfacial recombination loss and paves the way for a new strategy for tailoring HTLs for higher PSC performance.

*Doping level control on PEDOT:PSS—from highly doped to less doped*. To prepare dedoped PEDOT:PSS layers, we blend its solution with 4.5, 20, and 50% (vol %) of 1 M NaOH, which is then spin-coated onto different substrates for optical and electrical characterization as well as for devices.^30^ The normalized absorption spectra in Figure 1a show typical bipolaron peaks at wavelengths >1100 nm, which are reduced with an increasing NaOH blend ratio and accompanied by an increase in the polaronic peak at around 800 nm.^31,32^ The dedoping of PEDOT:PSS with NaOH is further confirmed by Raman spectroscopy (Figure 1b). The central peak around 1425 cm^–1^, assigned to the PEDOT intraring double bond oscillation, becomes narrower with a higher NaOH blend ratio.^32−34^ This correlates to the resonance of dedoped PEDOT with a 633 nm laser as can be seen from the absorption spectra. A decrease of the intraring single bond peak at 1370 cm^–1^ as well as the emergence of a new peak at 1520 cm^–1^ are consistent with previous reports of biased Raman spectroscopy of PEDOT:PSS.^35^ Note that beyond the 20% blend ratio, there is no significant change in the absorption or Raman spectra, indicating dedoping saturation is reached at the 20% NaOH blend ratio. The high blend ratio of 50% simply dilutes the PEDOT:PSS content, therefore reducing the absorbance and decreasing the signal-to-noise ratio.

**Figure 1:**
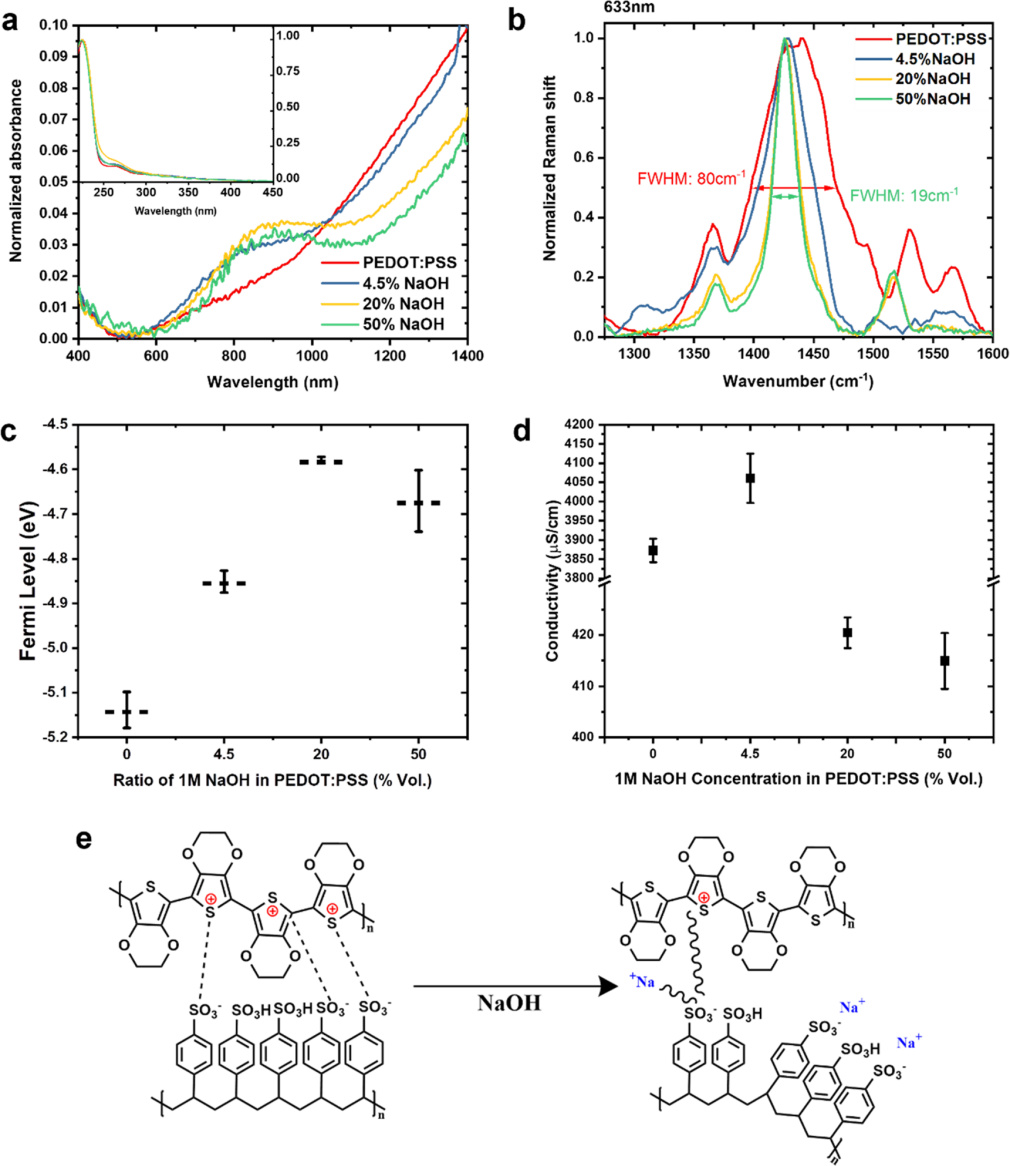
Optical and electrical properties of neat and dedoped PEDOT:PSS with 4.5, 20, and 50% volume fractions of 1 M NaOH. (a) UV–vis spectra normalized at 220 nm with the inset of full spectra, (b) Raman spectra with 633 nm laser excitation, (c) Fermi level (measured by Kelvin probe), (d) conductivity, and (e) dedoping mechanism.

The Fermi level (*E*_F_) of the blend series is measured by a Kelvin probe with respect to the vacuum level and, therefore, its magnitude is equivalent to the work function. It shows a clear linear trend, decreasing from −5.15 to −4.55 eV with an increasing NaOH ratio (Figure 1c). The *E*_F_ of the 4.5% blend (−4.85 eV) lies in between the neat and 20% blend samples, making it an ideal intermediate dedoping ratio for the study. On the other hand, the *E*_F_ of the 50% blend ratio has a similar *E*_F_ to the 20% blend but with a larger deviation. This could be possibly due to PSS dissociation in strong basic environment, which is in agreement with the lower absorbance and similar normalized Raman spectrum compared to the 20% blend sample (Figure 1a,b). For dedoped PEDOT:PSS, lower conductivity is measured as expected. Field effect transistor measurements reveal a significant drop in conductivity between the neat and 20% blend samples from around 3875 to 420 μS/cm (Figure 1d). The dedoping-induced change in conductivity is strongly correlated to the optical absorption. The main dedoping effect occurs between 4.5 and 20% NaOH, leading to a significant drop in the bipolaron absorption, accompanied by almost an order of magnitude drop in conductivity. An increase of NaOH from 20 to 50% does not produce any further changes in either absorption or conductivity, which is consistent with no significant changes in the Raman spectrum and the work function. Interestingly, however, the 4.5% blend shows a slightly higher conductivity compared to the neat sample, which suggests a potential contribution of the ionic current from sodium ions introduced by the NaOH solution. The mechanism of PEDOT:PSS dedoping by NaOH is illustrated in Figure 1e. The hydroxide ions deprotonate the acidic sulfonates and thus reduce the stabilization of the polarons by the sulfonate on PEDOT. These data show the possibility of fine-tuning the energetics of PEDOT:PSS by blending with NaOH solution, enabling further study of the influence of this on perovskite solar cells.

*Dedoped PEDOT:PSS HTL in perovskite devices*. To unveil the impact of dedoped PEDOT:PSS HTL on recombination losses and device characteristics, we apply neat, 4.5, and 20% blends in a p-i-n perovskite solar cell architecture: ITO/PEDOT:PSS/methylammonium lead iodide (MAPI)/phenyl-C61-butyric acid methyl ester (PCBM)/2,9-dimethyl-4,7-diphenyl-1,10-phenanthroline (BCP)/Ag, as shown in Figure 2a. From the neat to 20% blend, the short-circuit current density (*J*_SC_) and *V*_OC_ decrease simultaneously and, as a result, the overall device PCE decreases. However, we notice an improvement in the FF, originating mostly from a reduced shunt resistance (Figure S2b) as can be seen by the much shallower slope close to *J*_SC_ (Figure 2a). This indicates that the dedoped PEDOT:PSS HTL has better electron blocking capability, which is consistent with the widened bandgap shown in the absorption spectra (Figure 1a). Here, the highly doped PEDOT:PSS shows mainly a bipolaron absorption band (<1400 nm), and upon dedoping, this absorption band shifts to high energy (∼800 nm) due to more polarons, leading to a larger bandgap in the dedoped PEDOT:PSS. Dedoping, nonetheless, decreases the conductivity of PEDOT:PSS, which can slow down charge transport/extraction. Therefore, optimization of the HTL thickness is crucial to maintain efficient charge transport/extraction and, at the same time, utilize the benefits of reduced recombination and better charge selectivity. Herein, we control the HTL thickness by diluting the 20% blend PEDOT:PSS with deionized water to obtain a range of thickness from 80 ± 10 nm (no dilution) to 30 ± 10 nm (4 times dilution). Further dilution was attempted but failed to provide good coverage over the ITO substrates. Kelvin probe measurements confirm that the doping level is maintained under dilution (Figure S1**)** with *E*_F_ maintained at −4.58 ± 0.03 eV across different levels of dilution.

**Figure 2:**
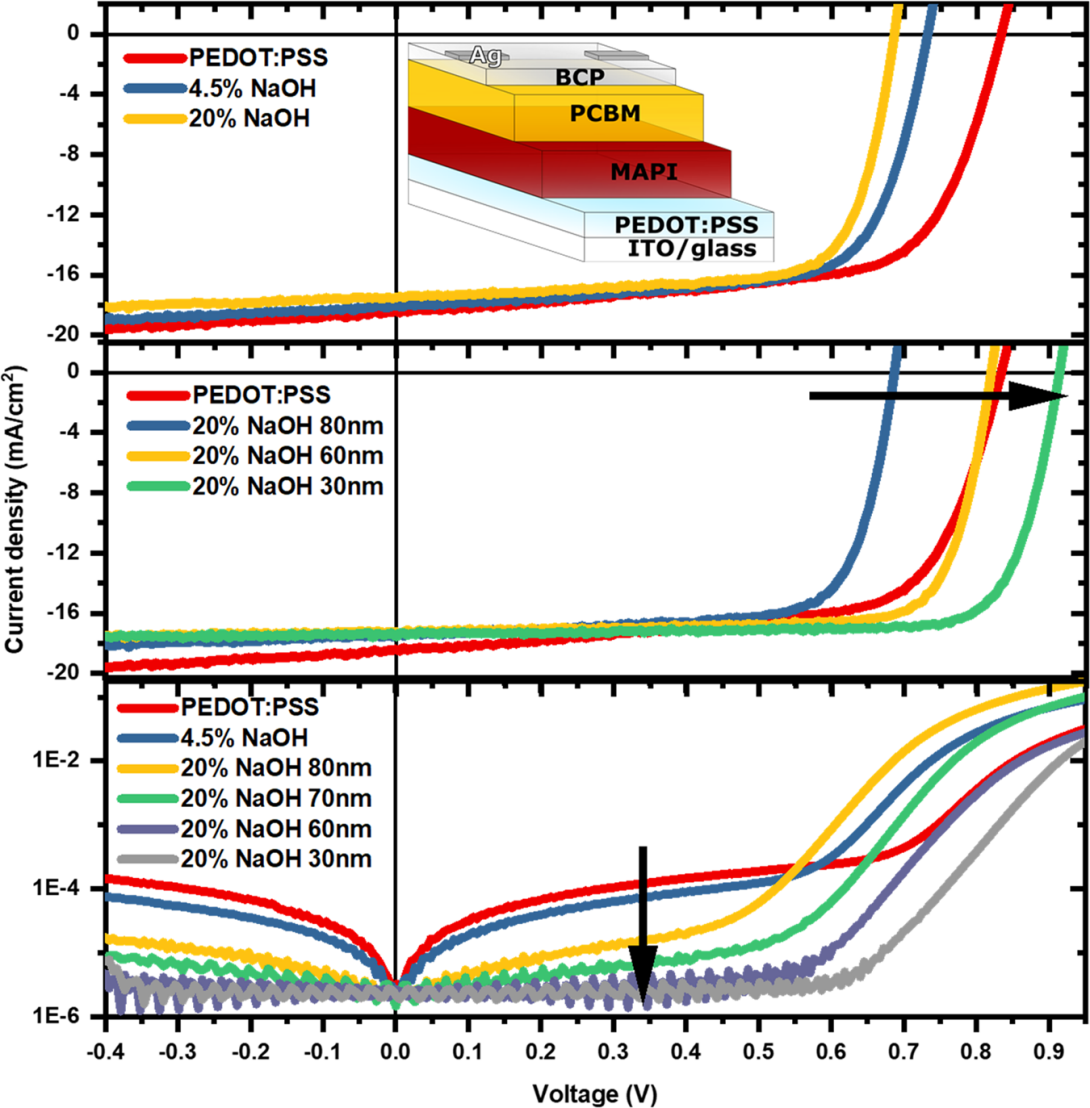
Device structure, performance. (a) Reverse current–voltage scans of NaOH-dedoped PEDOT:PSS devices, (b) reverse current–voltage scans of the 20% NaOH-dedoped PEDOT:PSS solar cells with different transport layer thicknesses, and (c) dark current–voltage scans of all NaOH-dedoped PEDOT:PSS devices.

With the optimized thickness, the device performance sharply increased as shown in Figure 2b with an average *V*_OC_ of 0.92 V and PCE of 12.7%. This is a 10% improvement for *V*_OC_ and 25% for PCE compared to the optimized neat PEDOT:PSS devices. The superior charge blocking ability can also be seen in the dark current–voltage scans (Figure 2c). Dark current in the shunt region (<0.2 V) is significantly reduced by dedoping, and the dark currents at 0.4 V (before turn-on) have been sharply reduced from 1.47 × 10^–3^ mA/cm^2^ (neat) to 3.05 × 10^–5^ mA/cm^2^ (20% blend, 30 nm). Note that the dark current density around 3 × 10^–5^ mA/cm^2^ is corresponding to the instrument sensitivity in the current (0.1 nA), so a plateau appears for the thin dedoped PEDOT:PSS devices. This indicates a significant increase in shunt resistance and a trend toward more-ideal diode behavior. Noticeably, the turn-on voltage is coherent with the *V*_OC_ trend, which decreases with increased dedoping of thick PEDOT:PSS layer and increases by reducing the thickness of dedoped PEDOT:PSS layer.

The statistics of *V*_OC_, *J*_SC_, shunt resistance, PCE, and FF are shown in Figures 3 and S2. The average *V*_OC_, as mentioned above, decreases from 0.83 to 0.69 V through dedoping due to the recombination of the accumulated charges at the interface and significantly improves to 0.92 V with a thinner HTL. Although thickness affects the charge extraction, dedoped PEDOT:PSS already improves the average FF from 0.67 to 0.74 without thickness optimization. Since their thickness is not optimized here, the improvement is mainly coming from the better charge blocking ability. With thickness optimization, the full potential of dedoping is utilized, and the average FF reaches 0.8 with our best devices. Surprisingly, the deviation of FF over different devices is reduced with dedoping. We believe this is due to a change in the wettability of the PEDOT:PSS surface, which results in better perovskite crystallization and higher consistency, as demonstrated by the AFM images in Figure S3d–f. Despite the huge drop in conductivity from dedoping (Figure 1d), only a small drop in the *J*_SC_ values is observed when dedoped PEDOT:PSS is incorporated into devices (Figure S2). As the dedoped PEDOT:PSS thickness is reduced, the *J*_SC_ improves slightly, consistent with the improvements in shunt resistance. Combining the improvement in FF and *V*_OC_, the overall PCE increased from 10.2% for the neat PEDOT:PSS devices to 12.7% for the optimized 30 nm 20% NaOH-dedoped devices.

**Figure 3:**
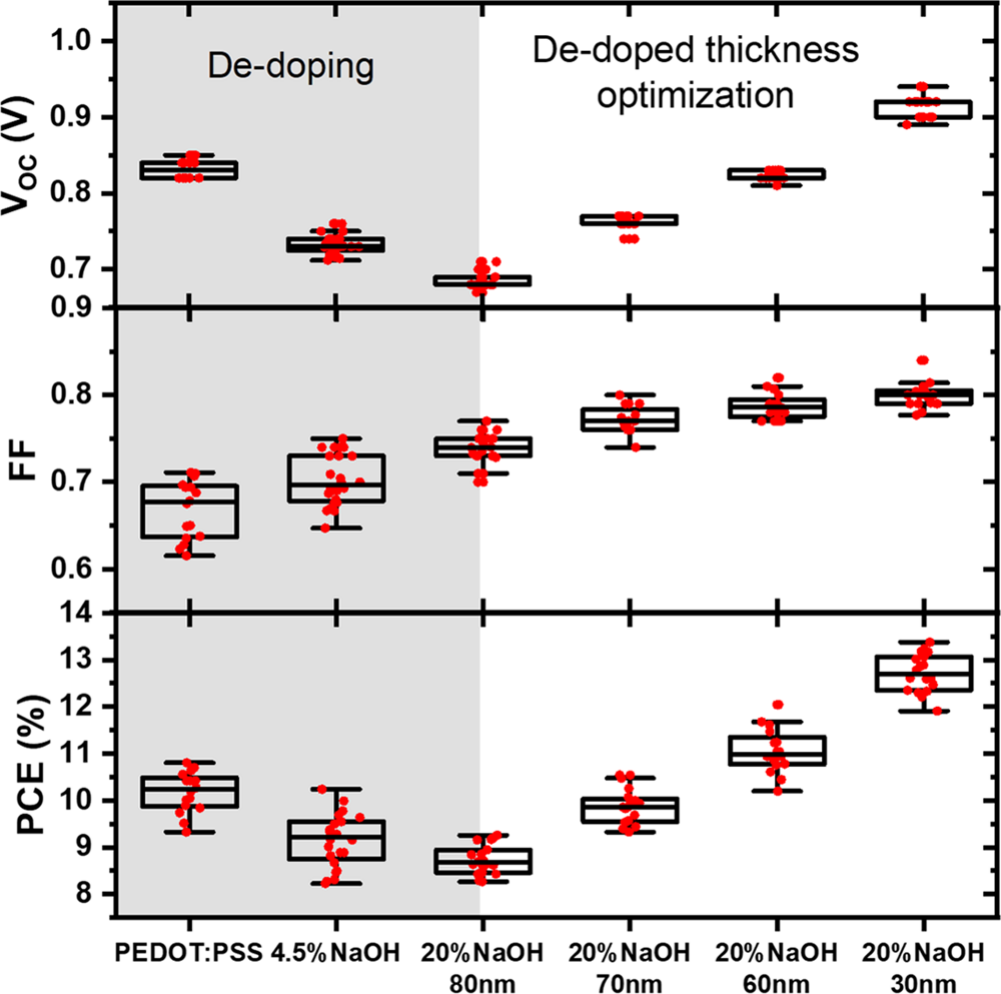
Box plots of open-circuit voltage (*V*_OC_), fill factor (FF), and power conversion efficiency (PCE) of NaOH-dedoped devices.

To confirm the origin of the performance improvement, we investigate the optoelectronic properties of half cells with a structure of ITO/HTL/MAPI (Figure 4). First, the intensity of photoluminescence (PL) of the MAPI layer is significantly enhanced when deposited on top of the dedoped PEDOT:PSS HTL layer and further increased when applying a thinner HTL layer. The higher magnitude of PL at open circuit (*i.e.*, no external connections) has been shown to indicate the strength of surface recombination loss.^9,36,37^ Since the half cells are not grounded, free charges generated by photoexcitation will remain in the system; thus, more nonradiative recombination of free charge carriers results in lower PL intensity. Notably, only a small PL shift (∼3 nm) occurs among different doping levels as seen in Figure S5, which is attributed to the small compositional differences across the MAPI films. We have shown that a small but similar amount of tail states is found among the perovskite layers on top of different dedoped PEDOT:PSS (Figure 4d). It indicates that even though tiny compositional difference may exist, the overall perovskite quality across samples is comparable, and the difference is negligible. Further evidence for reduced recombination losses can be seen from the transient PL probed at 780 nm (Figure 4b). The dedoped PEDOT:PSS samples show a slightly longer charge carrier lifetime even without thickness optimization, which is linked to reduced recombination loss at the interface. Nonetheless, the reduced recombination for thick PEDOT:PSS devices was not seen as an increase in *V*_OC_, due to the losses caused by its lower conductivity (Figure 2a). By decreasing the thickness of dedoped PEDOT:PSS, the reduced interfacial recombination loss is fully revealed and gives a nearly single exponential PL decay. This indicates that the radiative recombination dominates the system at optimal thickness. For comparison, we also measured the optical behaviors in full cells (Figure S6). With the top PCBM layer, the same trends in PL intensity and decay are observed; however, the charges generated by photoexcitation are fully extracted from perovskite layer, so a lower PL intensity and faster PL decay are observed, demonstrating a higher efficiency of charge extraction in the full device. The characteristic behavior from both PL intensity and dynamics suggests a reduction in nonradiative surface recombination with thin, dedoped PEDOT:PSS HTL.

**Figure 4:**
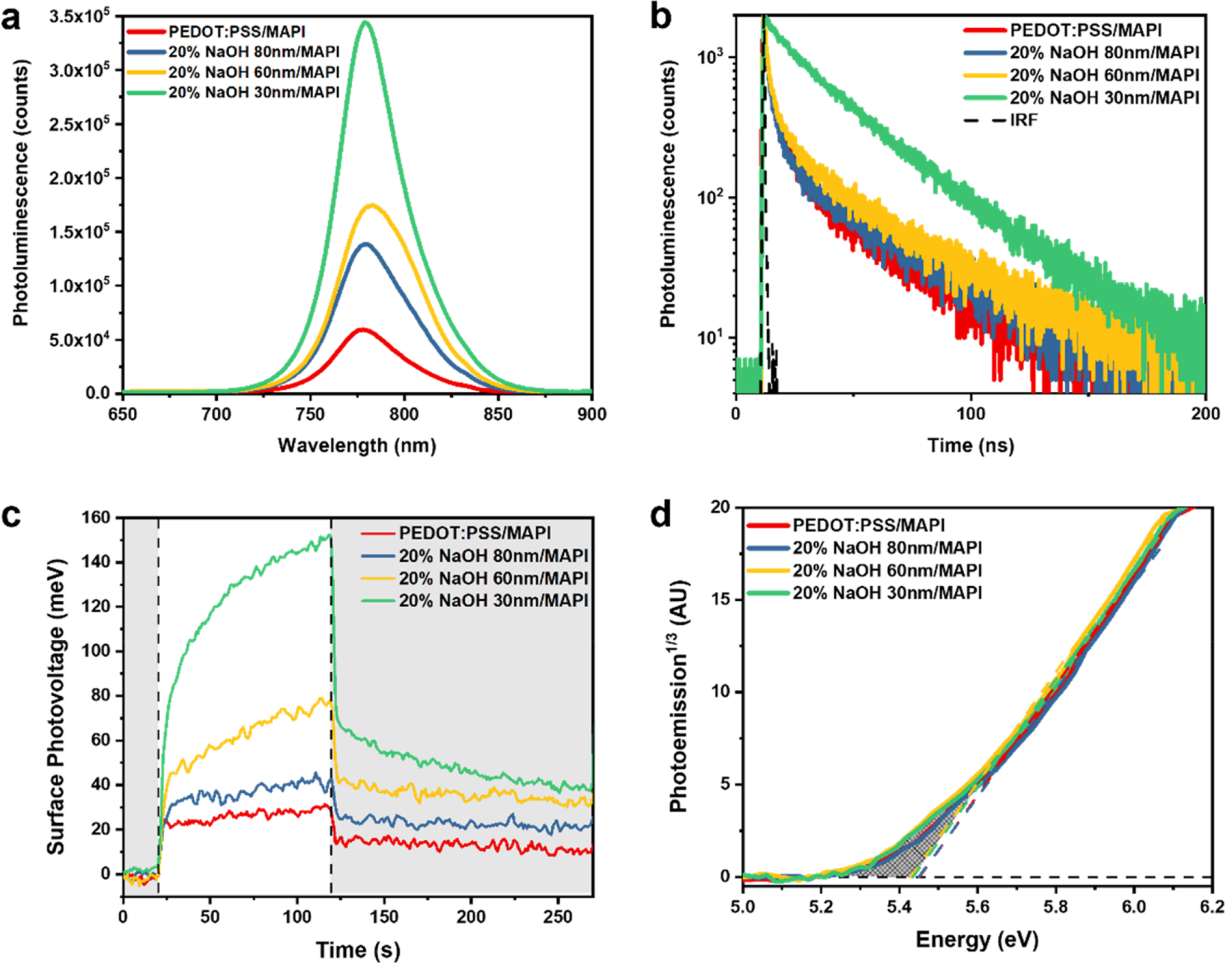
Thickness-dependent optical and optoelectronic measurements of NaOH-dedoped PEDOT:PSS HTL with MAPI on top. (a) Photoluminescence and (b) transient photoluminescence decay of MAPI probed at 780 nm (with a 405 nm excitation wavelength). (c) Surface photovoltage signals generated by 0.2 sun white light illumination. Dashed vertical lines indicate the transitions between light off (gray) and light on (white). (d) Ambient photoemission spectra of MAPI on top of doped and dedoped PEDOT:PSS HTL with different thicknesses.

To directly detect the differences in surface recombination between the doped and dedoped HTL, surface photovoltage (SPV) with MAPI on top was measured (Figure 4c).^27,38^ SPV measures the change of surface potential under illumination, which is related to the remaining charge density on the top surface and any ionic rearrangement within the perovskite. Since the samples are only grounded on the HTL side, the holes are fully extracted, while the electrons remain in the MAPI layer building up a surface voltage difference analogous to open-circuit conditions. The SPV signal is slightly larger with the 80 nm dedoped sample compared to the neat PEDOT:PSS, indicating a higher density of electrons accumulating in the MAPI layer. With thicker dedoped PEDOT:PSS devices, the advantage of dedoping is not clear in the device current–voltage curves due to the decreased conductivity opposing the increased selectivity and reduced recombination. However, when the thickness of dedoped PEDOT:PSS layer is reduced, a significantly larger SPV signal (+130 meV) is observed. The same results were seen in full cells using the optimized thickness of dedoped PEDOT:PSS producing the highest SPV signal (+80 meV) (Figure S6c). It is interesting to notice that the optimized dedoped PEDOT:PSS shows the largest but slowest SPV increase during illumination. This might indicate that with a larger amount of free charges in the perovskite layer, the strong electric field induced by the optimized HTL causes more ionic movement, resulting in slow turn-on in the SPV transient. As soon as illumination is off, there is a significant drop in SPV (<50% within the subsecond time scale), followed by a slow decay at longer time scale (<100 s). The main cause of this slow decay is not clearly understood at the moment, although any charge trapping induced by the photoactive layer (e.g., grain boundaries) cannot be ruled out, as found in organic bulk heterojunction blends (a large number of domains formed by donor and/or acceptor molecules) compared to bilayers (no domains formed).^39^ In the thicker dedoped PEDOT:PSS devices, the low conductivity of the HTL severely limits charge transport/extraction. With thickness optimization, this limitation on charge transport/extraction is less of an issue, and the SPV demonstrates that there is reduced recombination and improved charge selectivity at the HTL/MAPI interface.

In order to further confirm the changes are solely coming from a different doping level of the PEDOT:PSS layer, ambient photoemission spectroscopy (APS) measurements were performed for MAPI on top of different HTLs (Figures 4d and S4). The APS spectra of MAPI on top of all doped and dedoped HTLs show very similar signal intensity and extrapolated valence band edges (indicated by the dash line) as well as equal intraband tail states (indicated by the shaded area). In previous studies, the APS areas below the band edge were shown to be directly related to the trap states and defects in perovskites.^40−42^ Therefore, the same amount of tail states measured on all HTLs suggests that the physical and electronic composition of MAPI is unaffected by the different underlayer HTLs. Despite the similar amount of traps in the MAPI layer, atomic force microscopy (AFM) shows a significantly larger MAPI grain size when it is formed on top of dedoped PEDOT:PSS (Figure S3d–f). This is related to changes in PEDOT:PSS wettability due to NaOH quenching the sulfonate groups on the PSS units. With sodium quenching off the sulfonate group, the hydrophilicity of the polymer increases, which is beneficial to perovskite crystallization. This makes the perovskite layer fabrication more consistent, shown by reduced FF deviation between dedoped devices (Figure 3).

To illustrate the exceptionally low recombination loss mechanism revealed by the SPV measurements, a band alignment diagram is shown in Figure 5. At dark equilibrium, at the interface of PEDOT:PSS and MAPI there is downward band-bending in the MAPI (toward the surface) due to the shallower *E*_F_ of MAPI compared to PEDOT:PSS. The highly doped PEDOT:PSS has its *E*_F_ close to the HOMO level with a large density of pre-existing holes, which, under illumination, charge carriers are driven in opposite directions due to this interfacial band-bending. As charges accumulate at the PEDOT:PSS/MAPI interface, the band-bending is reduced toward open circuit. As this situation is reached, the excess holes in the PEDOT layer drive strong interfacial recombination, reducing the amount of electrons in the MAPI layer. Additionally, the highly doped PEDOT:PSS is less selective with a smaller bandgap and has more metallic character, which both contribute to interfacial recombination. These effects result in lower SPV and reduced *V*_OC_ in full devices. On the contrary, dedoped PEDOT:PSS has its *E*_F_ matching or slightly shallower than MAPI, resulting in a small, effectively negligible upward band-bending. This band-bending and the lower conductivity of dedoped HTL require a thinner transport layer for optimal hole extraction. With the optimized thickness, dedoped PEDOT:PSS benefits from the enhanced selectivity and reduced interfacial charge carrier recombination allowing for an increased density of electrons in the perovskite photoactive layer at the open-circuit condition. This is reflected in a sharp increase of recorded SPV signal and an increased *V*_OC_ and PCE of solar cells based on the thin, dedoped PEDOT:PSS HTL.

**Figure 5:**
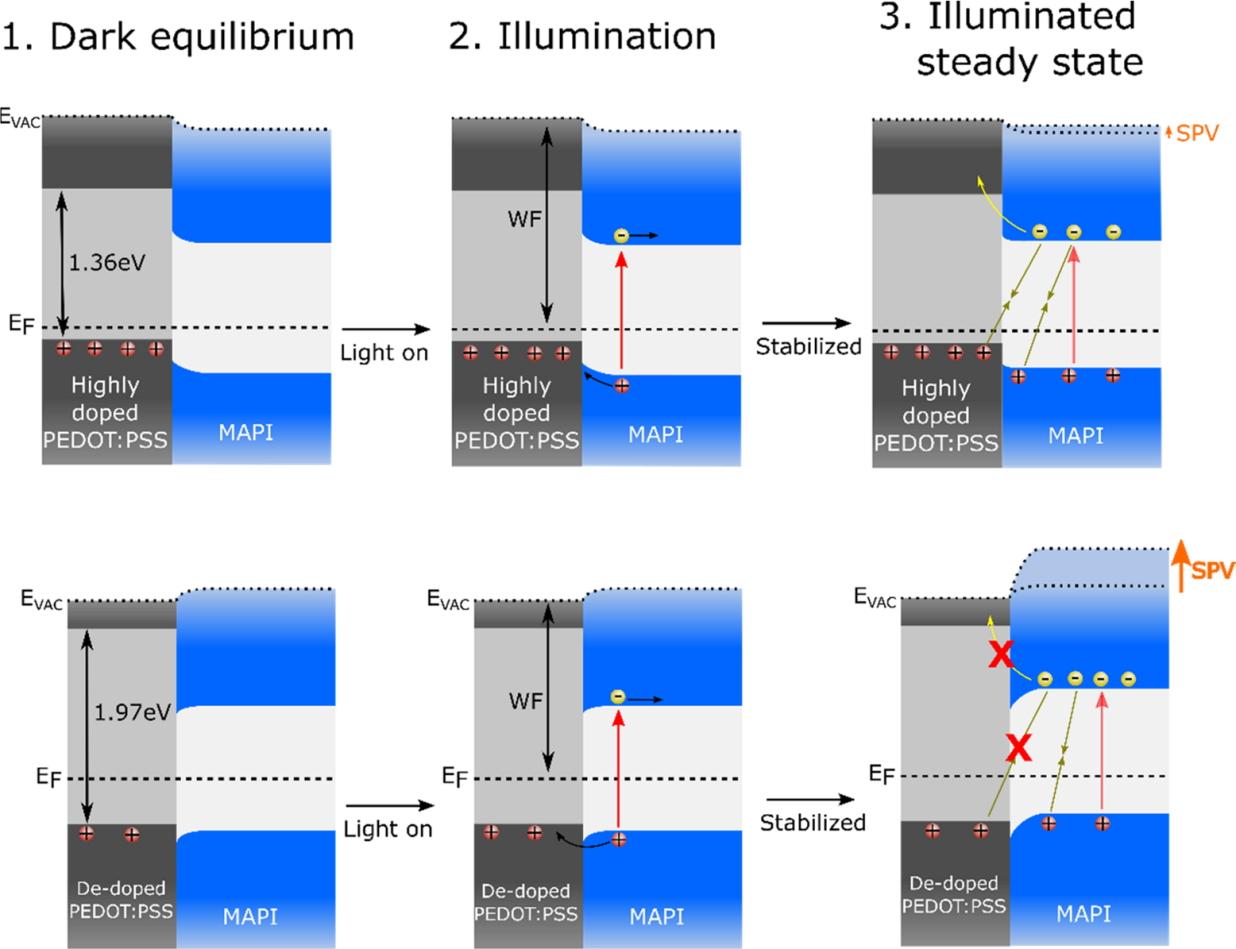
Schematic diagrams of surface photovoltage for neat and dedoped PEDOT:PSS with MAPI on top in three different stages. The Fermi level (*E*_F_) and local vacuum level (*E*_vac_) are indicated by dashed and dotted lines, respectively. The red arrows indicate charge generation, and the olive-green arrows indicate recombination. Measured SPV signals are shown by the orange arrow in the steady state stage. (Note that the confined interfacial band-bending in MAPI is drawn in a larger scale for visual clarity.)

In summary, this work demonstrates the correlation between the PEDOT:PSS doping level and interfacial recombination loss, providing a strategy to utilize less-doped HTLs in PSCs by thickness control. This approach should be applied to other HTL materials to confirm the generality of this strategy. Additionally, the higher performance from dedoping reveals another route to commercialize PSCs based on PEDOT:PSS HTL with its low cost and wide availability.

## Experimental Methods

### Device/Sample Fabrication

Substrates were either quartz or indium tin oxide (ITO)-coated glass depending on the type of measurement, with all substrates cleaned in an ultrasonic bath in 2% (v/v) Hellmanex III solution, deionized water, acetone, and isopropanol, consecutively. Then, 15 min of 100 W oxygen plasma treatment was performed before further deposition. Poly(3,4-ethylenedioxythiophene) polystyrenesulfonate (PEDOT:PSS, Clevios AI4083 Heraeus) was dedoped by 1 M sodium hydroxide solution by 4.5, 20, or 50% (v/v), and the well-mixed 20% blend solutions were then further diluted to 3:1, 1:1, and 1:3 volume ratios with deionized water. PEDOT:PSS solutions were then spin-coated at 2500 rpm for 1 min, followed by 10 min of annealing at 135 °C. Methylammonium lead iodide (MAPI) solutions (1.5 M) were prepared with lead iodide (Sigma-Aldrich, 99%) and methylammonium iodide (Sigma-Aldrich, 98%) and dissolved in a mix solution of DMF/DMSO (9:1.1,v/v). The MAPI layer was deposited by spin-coating at 4000 rpm and with 400 μL of diethyl ether dripped at 7 s. The samples were then annealed at 80 °C for 1 min and at 100 °C for 30 min. The electron transport layers were prepared by spin-coating [6,6]-phenyl-C61-butyric acid methyl ester (PCBM 98%, Ossila) from 23 mg/mL of chlorobenzene solution at 2000 rpm. Bathocuproine (BCP, Ossila) solution (0.05 mg/mL) was then spin-coated at 4000 rpm for 20 s to form a protective layer on top of PCBM. Finally, a 100 nm silver layer was evaporated as the anode contact.

### Absorption Spectroscopy and Raman Spectroscopy

Raman spectra were measured by a Renishaw inVia Raman microscope in a backscattering configuration. Measurements were done with a 633 nm helium–neon laser, and a 1200 line/mm diffraction grating was used. Averaging was implemented to improve the signal-to-noise ratio, and a polynomial background subtraction was carried out. Absorption spectra were measured with a Shimadzu UV-2600 UV–vis spectrophotometer. Absorbance was then obtained from the logarithm of the transmittance ratio between samples and a clean substrate.

### Energetics Measurements

Ambient photoemission spectroscopy (APS), Kelvin probe measurements, and surface photovoltage (SPV) measurements were taken using an APS04 from KP Technology. Before measuring test samples, the tip is calibrated by measuring the contact potential difference with respect to a silver reference. Then, the work function of the reference silver is determined by photoemission spectroscopy to calibrate the absolute work function of the tip with respect to the vacuum level. Test samples were grounded through the ITO for an unbiased electrical background. Measurements were done in the sequence of Kelvin probe, SP,V and APS to guarantee minimal degradation on the test sample by illuminations, and they were kept in the dark for around half an hour in order to reach dark equilibrium for initial Kelvin probe measurements. SPV illumination was with a quartz tungsten halogen light source (Dolan–Jenner) at ∼20 mW/cm^2^ intensity. A deuterium lamp was used to generate ultraviolet (UV) light for the APS measurements. The photoemitted free electrons and the radicals generated from them were collected by a positively biased tip. The cube root of the signal was then processed and linearly fitted to the HOMO level, and its tail state area was then integrated from the photoemission onset to the linear fitted region.^43^

### Conductivity

Conductivities of different dedoped PEDOT:PSS were measured using the transmission line method on organic field effect transistors with Fraunhofer prefabricated substrates.^44^ The conductance was extracted from output curves measured in four different channel lengths, 2.5, 5, 10, 20 μm, and the conductivity was derived from the slope of linear fitting.

### Emission Spectroscopy

Photoluminescence (PL) and transient PL were carried out with an FLS1000 photoluminescence spectrometer from Edinburgh Instruments. Samples were excited from the perovskite or PCBM top layer with 405 nm incident light from a xenon lamp. The emitted light was filtered by a 495 nm long-pass filter to eliminate interference from the incident light. Transient PL was done under the same filter setup with a 405 nm laser incident light source and with emission being measured at the main perovskite peak around 780 nm. The PL intensity was set to 2000 counts per second to provide a similar initial condition. The instrument response function was measured with cleaned quartz.

### Atomic Force Microscopy (AFM)

Surface topography was measured using a Park NX10 AFM with a PPP-NCHR type tip under noncontact mode. The images were aligned and calibrated with Gwyddion.

### Current–Voltage Characterization

The current–voltage characters of the devices were obtained using a solar simulator with AM1.5G filters (Oriel Instruments) and a Keithley 2400 Source Measure Unit. Calibration was done with a silicon photodiode (Osram BPW21). Devices were scanned from 1.2 to −0.4 V at a rate of 0.1 V/s.

## Supporting Information Available

Thin film energetics (*E*_F_, APS), solar cell performance (*J*_SC_, shunt resistance), AFM images, surface photovoltage (SPV), steady state photoluminescence, and transient photoluminescence (PDF)
