# Supplementary material for: Suppressing PEDOT:PSS Doping-Induced Interfacial Recombination Loss in Perovskite Solar Cells
Source: ACS Energy Lett. 2022 Jan 6;7(2):560–8. doi: 10.1021/acsenergylett.1c02577 (PMC9007524; doi:10.1021/acsenergylett.1c02577)
Supplement: Supplementary file 1 — nz1c02577_si_001.pdf [file nz1c02577_si_001.pdf]

Supporting information for Suppressing PEDOT:PSS Doping-Induced Interfacial Recombination Loss in Perovskite Solar Cells

*Yi-Chun Chin, Matyas Daboczi, Charlie Henderson, Joel Luke, Ji-Seon Kim**

Department of Physics and Center for Processable Electronics, Imperial College London, London SW7 2AZ, UK

AUTHOR INFORMATION

Corresponding Author

*Department of Physics and Center for Processable Electronics, Imperial College London, London SW7 2AZ, UK; orcid.org/0000-0003-4715-3656; Email: ji-seon.kim@imperial.ac.uk

Supporting Information

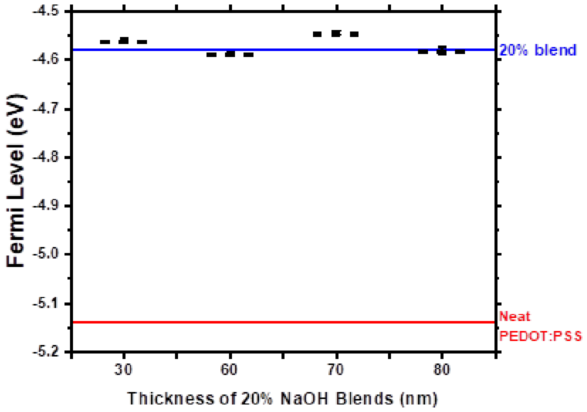

Figure S1. Fermi levels of 20 vol% NaOH de-doped PEDOT:PSS layers with different thicknesses

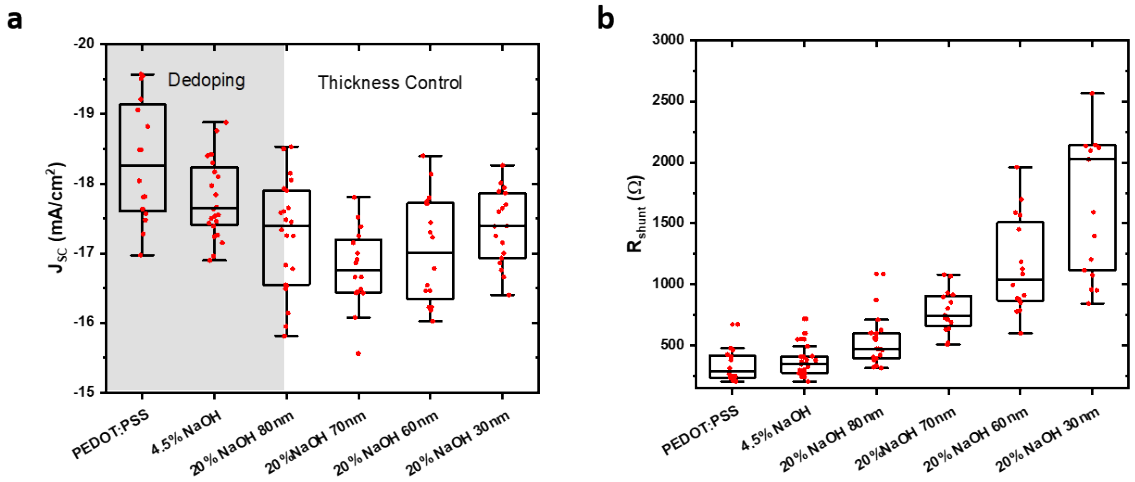

Figure S2. Statistics of (a) solar cell short-circuit current density (J_{SC}) and (b) shunt resistance (R_{Shunt})

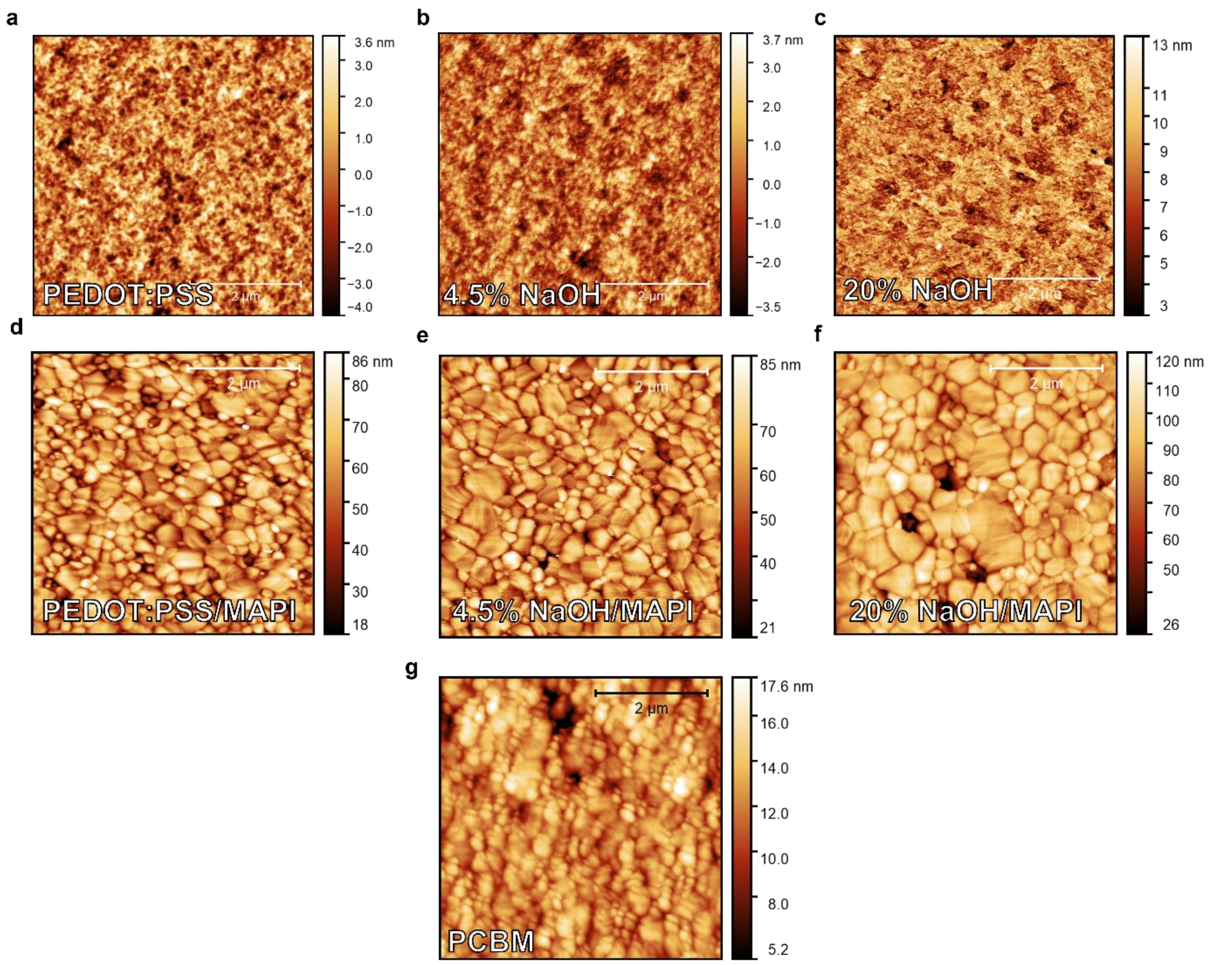

Figure S3. AFM image of PEDOT:PSS with different NaOH blend ratio in vol%, MAPI on top of PEDOT:PSS and PCBM on top of MAPI.

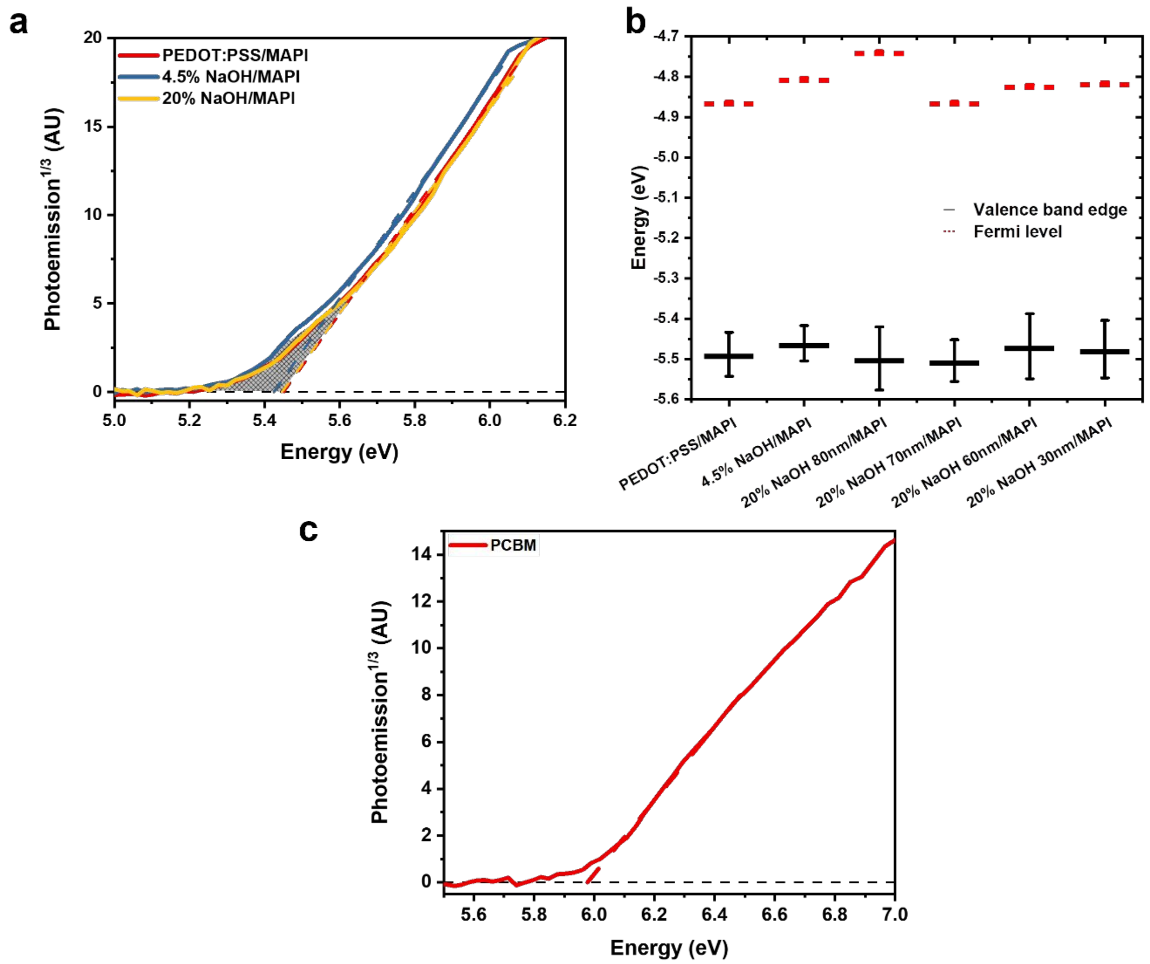

Figure S4. Energetics of MAPI and PCBM. (a) Ambient photoemission spectra of MAPI, and (b) valence band edge and Fermi levels of MAPI on top of neat, 4.5 vol%, and 20 vol% NaOH blend PEDOT:PSS. (c) Ambient photoemission spectrum of PCBM on top of MAPI.

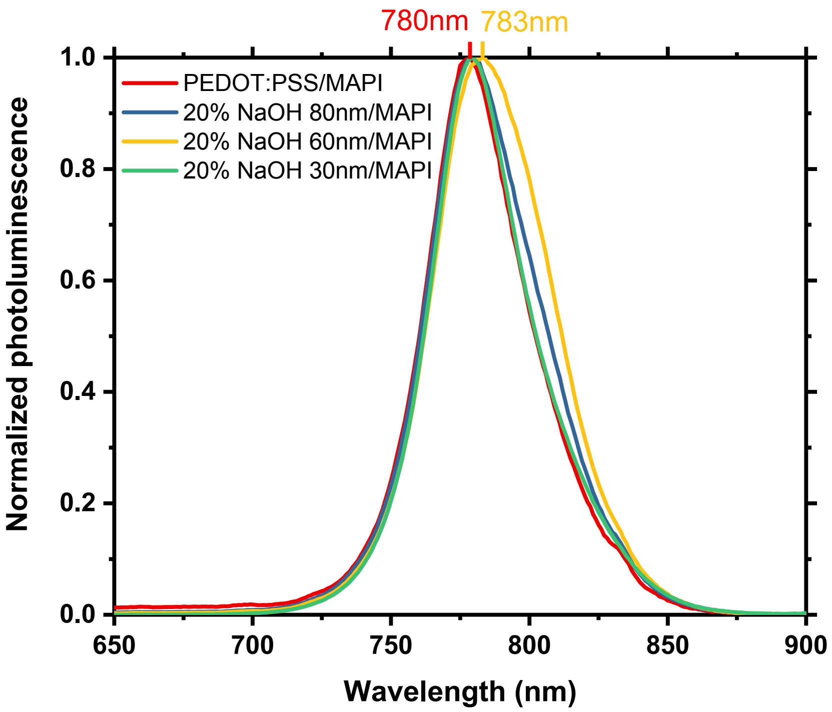

Figure S5. Normalized thickness dependent photoluminescence measurements of NaOH de-doped PEDOT:PSS HTL with MAPI on top probed with 405 nm excitation wavelength.

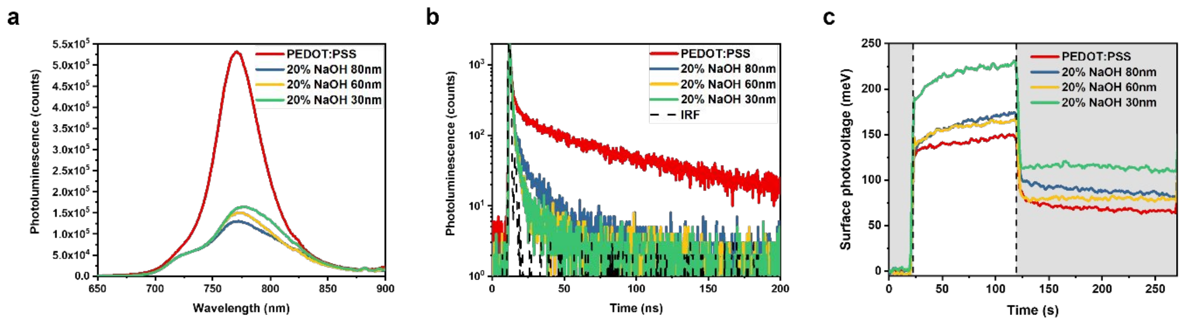

Figure S6. Thickness-dependent optical and optoelectronic measurements of NaOH de-doped PEDOT:PSS with MAPI and PCBM on top. (a) Photoluminescence and (b) transient photoluminescence decay with 405nm excitation. (c) Surface photovoltage generated by 0.2 sun white light illumination. Dashed vertical lines indicate the transitions between light off (grey) and light on (white).
